# Supplementary material for: Effects of pruning on tea tree growth, tea quality, and rhizosphere soil microbial community
Source: Microbiol Spectr. 2023 Sep 26;11(5):e01601-23. doi: 10.1128/spectrum.01601-23 (PMC10655597; doi:10.1128/spectrum.01601-23)
Supplement: Supplemental material — Tables S1 to S3. [file spectrum.01601-23-s0001.doc]

**Supplementary Materials**

**Table S1 35 genera of microorganisms with VIP > 1 were selected by OPLS-DA screening**

| **Gunes** | **SC-1** | **SC-2** | **SC-3** | **SP-1** | **SP-2** | **SP-3** | **SC VS SP** | **VIP > 1** |
| --- | --- | --- | --- | --- | --- | --- | --- | --- |
| *g__Acidobacterium* | 0.0140779 | 0.0144748 | 0.0110896 | 0.0042024 | 0.0048794 | 0.0026848 | down | 1.0220137 |
| *g__Acidocella* | 0.0003969 | 0.0004202 | 0.0004436 | 0 | 0 | 2.335E-05 | down | 1.0580468 |
| *g__Aquicella* | 0.0023113 | 0.0023813 | 0.0022179 | 0.0010272 | 0.0012841 | 0.0010506 | down | 1.0507107 |
| *g__Bacillus* | 0.0004903 | 0.0003502 | 0.0002802 | 4.669E-05 | 9.339E-05 | 4.669E-05 | down | 1.025105 |
| *g__Coxiella* | 0.0005136 | 0.000677 | 0.0004903 | 0.0001401 | 9.339E-05 | 7.004E-05 | down | 1.0360745 |
| *g__Granulicella* | 0.0055331 | 0.0056498 | 0.0042024 | 0.0016109 | 0.001821 | 0.001214 | down | 1.038819 |
| *g__Inquilinus* | 0.0014708 | 0.0018677 | 0.0012374 | 0.0003735 | 0.0004436 | 0.0001868 | down | 1.0067274 |
| *g__Mizugakiibacter* | 0.0016809 | 0.0017276 | 0.0020078 | 4.669E-05 | 4.669E-05 | 2.335E-05 | down | 1.0631557 |
| *g__Pedosphaera* | 0.0021012 | 0.0019611 | 0.0016343 | 0.0009572 | 0.0008872 | 0.0006304 | down | 1.0112403 |
| *g__Sporosarcina* | 0.0007938 | 0.0010973 | 0.0009339 | 7.004E-05 | 2.335E-05 | 4.669E-05 | down | 1.0430507 |
| *g__Acidicaldus* | 0.0043191 | 0.0053697 | 0.0052296 | 0.0110662 | 0.0107861 | 0.0081946 | up | 1.0104587 |
| *g__bacterium Ellin6543* | 0.0001401 | 9.339E-05 | 0.0001401 | 0.0008638 | 0.0010272 | 0.0008872 | up | 1.0576824 |
| *g__Blastochloris* | 0.0004903 | 0.0005837 | 0.0004436 | 0.0009572 | 0.0008872 | 0.0011206 | up | 1.0237622 |
| *g__Candidatus Microthrix* | 0 | 2.335E-05 | 0 | 0.0002335 | 0.0001401 | 0.0001401 | up | 1.0419905 |
| *g__Flavitalea* | 0.0001167 | 0.0001868 | 9.339E-05 | 0.0012374 | 0.0009105 | 0.0007004 | up | 1.0342786 |
| *g__Frankia* | 0.0001868 | 0.0001634 | 0.0002568 | 0.0005136 | 0.0005837 | 0.000537 | up | 1.0295006 |
| *g__Gaiella* | 0.0007938 | 0.0008171 | 0.000677 | 0.0018444 | 0.0017743 | 0.0016809 | up | 1.057422 |
| *g__Haliangium* | 0.0051596 | 0.005393 | 0.00586 | 0.0144048 | 0.0148951 | 0.013004 | up | 1.0612463 |
| *g__Iamia* | 4.669E-05 | 2.335E-05 | 4.669E-05 | 0.0002335 | 0.0004669 | 0.0004436 | up | 1.028446 |
| *g__Kaistia* | 0 | 0 | 2.335E-05 | 0.0001868 | 9.339E-05 | 0.0001401 | up | 1.0326026 |
| *g__Nakamurella* | 0 | 0 | 0 | 0.0001401 | 0.0001167 | 9.339E-05 | up | 1.063597 |
| *g__Nitrospira* | 0.0020078 | 0.0021012 | 0.0022179 | 0.0055098 | 0.0050195 | 0.0078444 | up | 1.0371219 |
| *g__Parafilimonas* | 7.004E-05 | 4.669E-05 | 7.004E-05 | 0.000537 | 0.0004436 | 0.000537 | up | 1.0596228 |
| *g__Pedomicrobium* | 0.0001634 | 0.0001401 | 0.0001167 | 0.0011673 | 0.0010973 | 0.0020311 | up | 1.0528768 |
| *g__Phaselicystis* | 0.0002101 | 0.0001634 | 0.0001868 | 0.0011907 | 0.0012841 | 0.0015409 | up | 1.0640506 |
| *g__Pseudonocardia* | 4.669E-05 | 0 | 2.335E-05 | 0.0003735 | 0.0003269 | 0.0002101 | up | 1.0098887 |
| *g__Reyranella* | 0.0022646 | 0.0021012 | 0.0027782 | 0.0059534 | 0.007611 | 0.0052296 | up | 1.0244053 |
| *g__Rhizorhapis* | 0 | 0 | 0 | 9.339E-05 | 4.669E-05 | 9.339E-05 | up | 1.0412154 |
| *g__Rhodoblastus* | 0 | 0 | 0 | 0.0007704 | 0.0008171 | 0.0014942 | up | 1.0658225 |
| *g__Rhodococcus* | 2.335E-05 | 2.335E-05 | 2.335E-05 | 0.0002335 | 0.0003269 | 0.0001634 | up | 1.0512887 |
| *g__Rhodoplanes* | 0.0002335 | 0.0002335 | 0.0003969 | 0.0013541 | 0.0011673 | 0.0012607 | up | 1.0399236 |
| *g__Sphingomonas* | 0.0012374 | 0.0012374 | 0.0013307 | 0.0019845 | 0.0019144 | 0.0017043 | up | 1.0306482 |
| *g__Sphingopyxis* | 0.0001634 | 0.0002335 | 0.0002335 | 0.0013541 | 0.001144 | 0.0011206 | up | 1.0578698 |
| *g__Verrucomicrobia bacterium* | 0 | 0 | 0 | 0.0001401 | 7.004E-05 | 0.0001634 | up | 1.046643 |
| *g__Woodsholea* | 2.335E-05 | 4.669E-05 | 2.335E-05 | 0.0002568 | 0.0002568 | 0.0003502 | up | 1.0456469 |

**Table S2 38 microbial metabolic pathways with VIP > 1 were obtained by OPLS-DA screening**

| **ID** | **KEGG_Pathways3** | **SC-1** | **SC-2** | **SC-3** | **SP-1** | **SP-2** | **SP-3** | **SC VS SP** | **VIP > 1** |
| --- | --- | --- | --- | --- | --- | --- | --- | --- | --- |
| ko00030 | Pentose phosphate pathway | 28509.6 | 28684.46 | 28149.45 | 27356.95 | 27648.12 | 27387.64 | down | 1.048396 |
| ko00052 | Galactose metabolism | 14841.56 | 14840.19 | 14278.09 | 12920.55 | 13393.34 | 13260.28 | down | 1.058322 |
| ko00450 | Selenocompound metabolism | 29311.02 | 29397.4 | 28756.95 | 28198.41 | 28159.59 | 28306.86 | down | 1.015156 |
| ko00500 | Starch and sucrose metabolism | 15732.35 | 15903.25 | 15241.96 | 14456.86 | 14774.28 | 14440.18 | down | 1.039153 |
| ko00510 | N-Glycan biosynthesis | 1860.104 | 1832.876 | 1837.325 | 1736.41 | 1746.372 | 1733.966 | down | 1.065038 |
| ko00511 | Other glycan degradation | 10008.52 | 9864.835 | 9036.876 | 6767.385 | 7344.109 | 6748.563 | down | 1.07001 |
| ko00520 | Amino sugar and nucleotide sugar metabolism | 18547.67 | 18651.71 | 18245.81 | 17513.26 | 17717.39 | 17772.41 | down | 1.041832 |
| ko00521 | Streptomycin biosynthesis | 37184.39 | 37255.34 | 36447.64 | 35060.61 | 35471.17 | 35645.19 | down | 1.033408 |
| ko00531 | Glycosaminoglycan degradation | 3463.879 | 3468.869 | 3149.926 | 2354.164 | 2672.443 | 2451.33 | down | 1.059892 |
| ko00540 | Lipopolysaccharide biosynthesis | 30119.91 | 29511.42 | 28909.78 | 27176.06 | 26814.62 | 27556.46 | down | 1.02195 |
| ko00600 | Sphingolipid metabolism | 4205.114 | 4085.214 | 3827.529 | 3110.767 | 3359.637 | 3072.293 | down | 1.060475 |
| ko00983 | Drug metabolism - other enzymes | 18409.36 | 18440.28 | 18060.72 | 17191.14 | 17325.04 | 17649.15 | down | 1.012881 |
| ko01055 | Biosynthesis of vancomycin group antibiotics | 59870.06 | 58139.83 | 56277.23 | 51105.13 | 51645.32 | 52491.9 | down | 1.043295 |
| ko02040 | Flagellar assembly | 32429.93 | 32124.2 | 30616.78 | 29259.53 | 29142.43 | 28844.41 | down | 1.028319 |
| ko03015 | mRNA surveillance pathway | 19.2778 | 17.3519 | 18.1296 | 7.0926 | 6.4815 | 5.1481 | down | 1.047871 |
| ko03022 | Basal transcription factors | 58.6154 | 52.3846 | 54.4615 | 19.8462 | 18.6923 | 12.4615 | down | 1.039013 |
| ko04142 | Lysosome | 718.195 | 722.3824 | 681.3918 | 540.8594 | 587.378 | 537.3846 | down | 1.064464 |
| ko00072 | Synthesis and degradation of ketone bodies | 32141.8 | 34641.05 | 34510.56 | 39311.76 | 39598 | 37803.62 | up | 1.003345 |
| ko00310 | Lysine degradation | 10570.72 | 11348.39 | 11457.98 | 12918.6 | 13011.74 | 12545.27 | up | 1.014086 |
| ko00312 | beta-Lactam resistance | 401.2575 | 445.135 | 438.5775 | 521.7125 | 513.0925 | 499.5875 | up | 1.015395 |
| ko00362 | Benzoate degradation | 7656.567 | 8312.65 | 8326.588 | 9658.784 | 9779.719 | 9234.477 | up | 1.004458 |
| ko00363 | Bisphenol degradation | 8658.455 | 9706.73 | 9700.132 | 11800.94 | 11935.14 | 11097.18 | up | 1.001708 |
| ko00410 | beta-Alanine metabolism | 16384.24 | 17503.83 | 17504.19 | 19701.3 | 19945.2 | 19065.68 | up | 1.005499 |
| ko00472 | D-Arginine and D-ornithine metabolism | 8073.69 | 9107.35 | 9183.62 | 12305.49 | 11298.35 | 11711.87 | up | 1.049673 |
| ko00473 | D-Alanine metabolism | 30287.86 | 31042.62 | 30862.56 | 32794.85 | 32391.85 | 32450.25 | up | 1.045929 |
| ko00591 | Linoleic acid metabolism | 3751.747 | 4246.885 | 4253.75 | 5290.025 | 5216.707 | 5050.613 | up | 1.026303 |
| ko00621 | Dioxin degradation | 2496.863 | 2886.79 | 2934.913 | 3857.971 | 3783.417 | 3875.961 | up | 1.046193 |
| ko00622 | Xylene degradation | 1258.845 | 1497.448 | 1494.578 | 1840.603 | 1883.334 | 1861.602 | up | 1.009018 |
| ko00630 | Glyoxylate and dicarboxylate metabolism | 19176.52 | 20095.02 | 20154.63 | 21792.47 | 21521.42 | 21554.78 | up | 1.032526 |
| ko00633 | Nitrotoluene degradation | 8683.529 | 9227.347 | 9409.613 | 10481.81 | 10413.79 | 10026 | up | 1.002384 |
| ko00642 | Ethylbenzene degradation | 6059.32 | 6476.548 | 6554.51 | 7328.55 | 7428.003 | 7178.512 | up | 1.016355 |
| ko00680 | Methane metabolism | 7436.923 | 7751.388 | 7738.951 | 8264.304 | 8243.347 | 8112.389 | up | 1.007787 |
| ko00750 | Vitamin B6 metabolism | 22208.93 | 22741.15 | 22766.27 | 23874.33 | 23781.81 | 23390.54 | up | 1.000582 |
| ko00780 | Biotin metabolism | 19746.04 | 20847.29 | 20932.29 | 24195.7 | 24293.37 | 23297.75 | up | 1.031449 |
| ko00791 | Atrazine degradation | 3795.511 | 4315.246 | 4282.471 | 5221.277 | 5038.967 | 4944.107 | up | 1.011003 |
| ko00980 | Metabolism of xenobiotics by cytochrome P450 | 11294.32 | 12495.25 | 12344.14 | 14870.1 | 14394.02 | 13843.38 | up | 1.004171 |
| ko02010 | ABC transporters | 14231.32 | 15417.08 | 15301.53 | 17089.34 | 16756.79 | 16661.71 | up | 1.004801 |
| ko03013 | RNA transport | 466.2988 | 467.7959 | 474.0202 | 520.9926 | 505.9285 | 496.1286 | up | 1.015765 |

**Table S3 Basic conditions of tea plantation during the experiment**

| Maximum temperature (℃) | Minimum temperature (℃) | Mean temperature (℃) | Number of sunny days (Day) | Precipitation days (Day) | Amount of precipitation (mm) | Relative humidity (%) | Pest and disease control | The pest and disease control in tea plantation was based on organic pesticides, the main organic pesticides used were matrine and azadirachtin, each organic pesticide was used three times a year in March, September and October, two organic pesticides were used alternately, and the use time interval of 10 days. |
| --- | --- | --- | --- | --- | --- | --- | --- | --- |
| Aug-21 | 35 | 23 | 27.7 | 13 | 18 | 194.5 | 79 |
| Sep-21 | 36 | 20 | 27.4 | 21 | 9 | 13.6 | 71 |
| Oct-21 | 33 | 11 | 20.9 | 17 | 14 | 35.6 | 73 |
| Nov-21 | 23 | 3 | 14 | 18 | 12 | 77.3 | 76 |
| Dec-21 | 20 | 2 | 10 | 22 | 9 | 32.7 | 71 |
| Jan-22 | 20 | 1 | 9.7 | 14 | 17 | 54.2 | 80 |
| Feb-22 | 23 | 1 | 7.6 | 10 | 18 | 151 | 82 |
| Mar-22 | 27 | 3 | 16.1 | 13 | 18 | 309.9 | 79 |
| Apr-22 | 33 | 6 | 18 | 13 | 17 | 112.6 | 74 |
